# Effect of houttuynia on improving lung injury in chronic obstructive pulmonary disease by regulating the TLR4 signaling pathway

**DOI:** 10.1002/fsn3.1922

**Published:** 2021-06-01

**Authors:** Wei Wang, Wei Wu, Bin Wang, Feng Gao

**Affiliations:** ^1^ Department of Respiratory Wangjing Hospital Chinese Academy of Traditional Chinese Medicine Beijing China

**Keywords:** chronic obstructive pulmonary, houttuynia, MyD88, TLR4

## Abstract

This study aimed to investigate the effects and mechanisms of houttuynia on lung pathological injury in rats with chronic obstructive pulmonary disease (COPD). Rats were randomly divided into the normal control, COPD model (model), low‐dose treatment (low, 5 mg/kg), middle‐dose treatment (middle, 10 mg/kg), and high‐dose treatment (high, 25 mg/kg) groups. The COPD rat model was induced by smoking combined with intratracheal instillation of lipopolysaccharide. The treatment groups were given *Houttuynia* by gavage at 30 min before smoking. The IL‐6, IL‐1β, and TNF‐α concentrations in serum and BALF were determined by ELISA. The pathological morphology was detected by HE staining. The apoptosis cell number was evaluated by TUNEL assay. Apoptotic proteins (caspase‐3 and caspase‐9) were measured by IHC assay in lung tissues. The relative proteins [TLR4, MyD88, and p‐NF‐κB(p65)] were evaluated by Western blot assay in lung tissues. Compared with the model group, the low, middle, and high groups could reduce pulmonary congestion, edema, inflammatory cell infiltration, and apoptosis cell number; downregulate the protein expression of caspase‐3, caspase‐9, TLR4, MyD88, and NF‐κB(p65) (*p* < .05); and inhibit the IL‐6, IL‐1β, and TNF‐α concentrations in serum and BALF. *Houttuynia* could improve the morphology and apoptosis cell number in lung tissues, thereby inhibiting the activation of the TLR4/MyD88/NF‐κB(p65) signaling pathway.

## Graphical abstract

In this study, we clearly discuss houttuynia's effects in COPD treatment and relative mechanisms by vivo study.

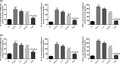

## 1 INTRODUCTION

Chronic obstructive pulmonary disease (COPD) is a disease resulting from chronic abnormal pulmonary inflammatory response induced by various reasons, and it is influenced by genetic and environmental factors. COPD is characterized by progressive aggravation and incompletely reversible airflow obstruction (Steidl et al., 2015; Vestbo et al., 2013). At present, tobacco smoking is believed to be an important inducer of COPD. Long‐term inflammatory stimulation may cause the infiltration of inflammatory cells (e.g., neutrophils, lymphocytes, and macrophages) and then release multiple inflammatory cytokines to participate in the pulmonary inflammatory response (Sarkar et al., 2015). Host defense can be divided into innate and adaptive immune system responses. The innate immune system can react rapidly through the interaction of Toll‐like receptors with highly conserved molecules in microorganisms. For example, TLR4 recognizes the lipopolysaccharide (LPS) of Gram‐negative bacteria and activates the nuclear factor‐κB (NF‐κB) signaling pathway, which is a key medium of the inflammatory response, after binding with its epitope; it then regulates the expression of cytokines, chemokines, and pro‐inflammatory genes (Kuzmich et al., 2017; Yaghchiyan et al., 2019). Meanwhile, TLR4 and NF‐κB(p65) are abnormally activated in patients with COPD (Pace et al., 2013; Rom et al., 2013; Venardos et al., 2014).

The cordate houttuynia is the fresh whole plant or the dry aerial part of the saururaceae plant *Houttuynia cordata* Thunb, which belongs to traditional Chinese medicine. Cordate houttuynia has the effect of clearing away heat and detoxifying. It is mainly used to treat pulmonary carbuncle, pyemesis, phlegmatic heat, asthmatic cough, heat dysentery, pyretic stranguria, abscess, swelling, and sores. Its therapeutic mechanism is mainly related to its antipathogenic microorganism, anti‐inflammatory, regulating immune function, and other pharmacological effects (Woranam et al., 2020). However, its injection can cause serious allergic reactions that restrict its full application. Houttuynin is one of the main components of the cordate houttuynia, which has sterilization, and antivirus and antitumor activities. It can inhibit cell inflammation and regulate immunity through the NF‐κB inflammatory pathway (Gao et al., 2014; Murray et al., 2006). In this study, the rat model of COPD was induced by smoking combined with intratracheal instillation of LPS to explore the regulatory effect and mechanism of houttuynin on pulmonary pathological injury, oxidative stress, and the TLR4/NF‐κB(p65) signaling pathway. This study is expected to provide experimental data for the clinical treatment of COPD.

## 2 MATERIALS AND METHODS

### 2.1 Experimental animals

Forty‐five six‐week‐old male SD rats were randomly divided into the normal control group (NC), COPD model group (model), low‐dose treatment group (low, model + 5 mg/kg houttuynin), middle‐dose treatment group (middle, model + 10 mg/kg houttuynin), and high‐dose treatment group (high, model + 25 mg/kg houttuynin). The average weight of rats (Certificate No.: SCXK (Beijing) 2019‐0001) was 200 ± 20 g, which were purchased from Beijing Vital River Laboratory Animal Technology Co., Ltd. The rats were fed at 26–28°C and exposed to light for 12 hr, with free access to food and water. The experiment was carried out 1 week after adaptive feeding.

### 2.2 Reagents and instruments

**FIGURE 1:**
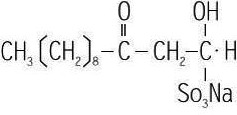
The structure of houttuynin

The structure of houttuynin is shown in Figure 1. Houttuynia sodium (Shanghai Qingping Pharmaceutical Co., Ltd., Batch No.: 1306‐5 and mass fraction of ≥97%), LPS (Sigma); Tianxiaxiu cigarettes (Sichuan China Tobacco Industry Co., Ltd.); HE staining kit (Nanjing Jiancheng Bioengineering Research Institute); monoclonal antibody (Abcam); ECL high‐sensitivity luminescent liquid (Invitrogen); TNF‐α, IL‐1β and IL‐6 ELISA kits (RB); BCA protein quantitative kit (Beyotime Institute of Biotechnology); Gel View 6000 gel imaging system (Guangzhou Yunxing Instrument Co., Ltd.); multifunctional Microplate Reader (TECAN); and general optical microscope (Olympus) were used in this study.

## 3 METHODS

### 3.1 Modeling

A rat model of COPD was established by smoking combined with intratracheal instillation of LPS. At 1 and 14 days, 200 μl (1 g/L) of LPS was dripped into the trachea. At 2–13 and 15–42 days, smoking was administered twice: one in the morning and one in the afternoon. The interval time was more than 6 hr, with 10 smokes per time and each time for 1 hr (Miao et al., 2015). In the low, middle, and high groups, corresponding doses of houttuynin (5, 10, and 25 mg/kg, respectively) were given by gavage administration at 30 min before the morning smoking at 15 days. The NC group and model group were given the same amount of normal saline by gavage. The treatment in all groups lasted for 4 weeks. At 24 hr after the last tracheal instillation, the five groups of rats were anaesthetized by intraperitoneal injection of chloral hydrate and fixed on the experimental animal platform. The abdomen was opened and about 5 ml of blood was collected from the abdominal aorta. The blood samples were centrifuged at 2,000 *g* and 4°C for 20 min, and the serum was collected and stored in a refrigerator at −80°C for testing. After blood collection, the trachea was exposed and the right lung was clamped, followed by the placement of the indwelling needle. The left whole lung was lavaged by using normal saline (2 ml each time) and the irrigation solution was fully collected, followed by another three times of repeated lavage. A recovery rate of more than 2/3 indicated a successful lavage. The irrigation solution was centrifuged at a centrifugal speed of 2,000 rpm at 20°C for 20 min, and the supernatant was retained and stored in the refrigerator at −80°C. The right lung of rats was fixed in 0.1 mol/L polyformaldehyde for histopathological analysis.

### 3.2 HE staining

The left lung was fully washed with precooled PBS and fixed with 10% neutral formalin, followed by dehydration, transparent processing, waxing, and embedding. The tissue was cut to about 5‐μm‐thick sections. HE staining was performed to observe the pathological changes and fibrosis of the lungs under the optical microscope.

### 3.3 Detection of cell apoptosis

TUNEL assay was used to detect the apoptosis of pulmonary vascular endothelial cells. The procedure was carried out according to the instructions of the kit, and positive nuclear staining was dark brown. Three sections were obtained from each lung tissue sample. Ten different visual fields were selected from each section, and the number of TUNEL‐positive cells was counted.

### 3.4 Immunohistochemistry (IHC)

The paraffin‐embedded sections (5 μm) of the left lung of rats were added with the primary antibodies of caspase‐3 and caspase‐9 and incubated overnight at 4°C. Biotinylated goat anti‐rabbit secondary antibody solution was added for reaction, followed by DAB development and hematoxylin re‐staining of the cell nucleus. The sections were then dehydrated with ethanol, sealed with neutral gum, and observed under a microscope. ImageJ image analysis software was used to analyze the expression of related proteins.

### 3.5 Western blot (WB) detection

The right lung tissue was cut into pieces and added with lysate for lysis. The homogenate was centrifuged for 10 min and the supernatant was separated, followed by the measurement of the protein concentration at a wavelength of 562 nm. With the preparation of the SDS‐PAGE gel, 10 μl of protein was loaded on each well. After electrophoresis, the gel within the range of the target protein was cut off and the protein was transferred to the NC membrane and sealed with 5% skim milk for 2 hr. After elution with PBS‐T buffer for three times (3 min each time), the sample was added with primary antibody and incubated at 4°C. It was then eluted with PBS‐T for three times (3 min each time). Subsequently, the secondary antibody was added to incubate for 2 hr, followed by another round of PBS‐T buffer elution for three times (3 min each time). After ECL development, the gel imager was used for exposure and development.

### 3.6 Detection of TNF‐α, IL‐1β, and IL‐6 by ELISA

The contents of TNF‐α, IL‐1β, and IL‐6 were detected in serum and bronchoalveolar lavage fluid (BALF) of rats in each group. The specific operation was carried out in accordance with the instructions of the ELISA kit. The absorbance at 450 nm (A450) was measured with a broad‐spectrum microplate reader. The standardized curve was drawn, and the contents of TNF‐α, IL‐1β, and IL‐6 were calculated.

### 3.7 Statistical analysis

SPSS 20.0 software was used for statistical analysis, and experimental data were expressed by mean ± *SD*. The data between the two groups were analyzed by independent‐samples *t* test, and the data among multiple groups were compared by one‐way analysis of variance.

## 4 RESULTS

### 4.1 Effect of houttuynin on the content of inflammatory cytokines in COPD rats

**FIGURE 2:**
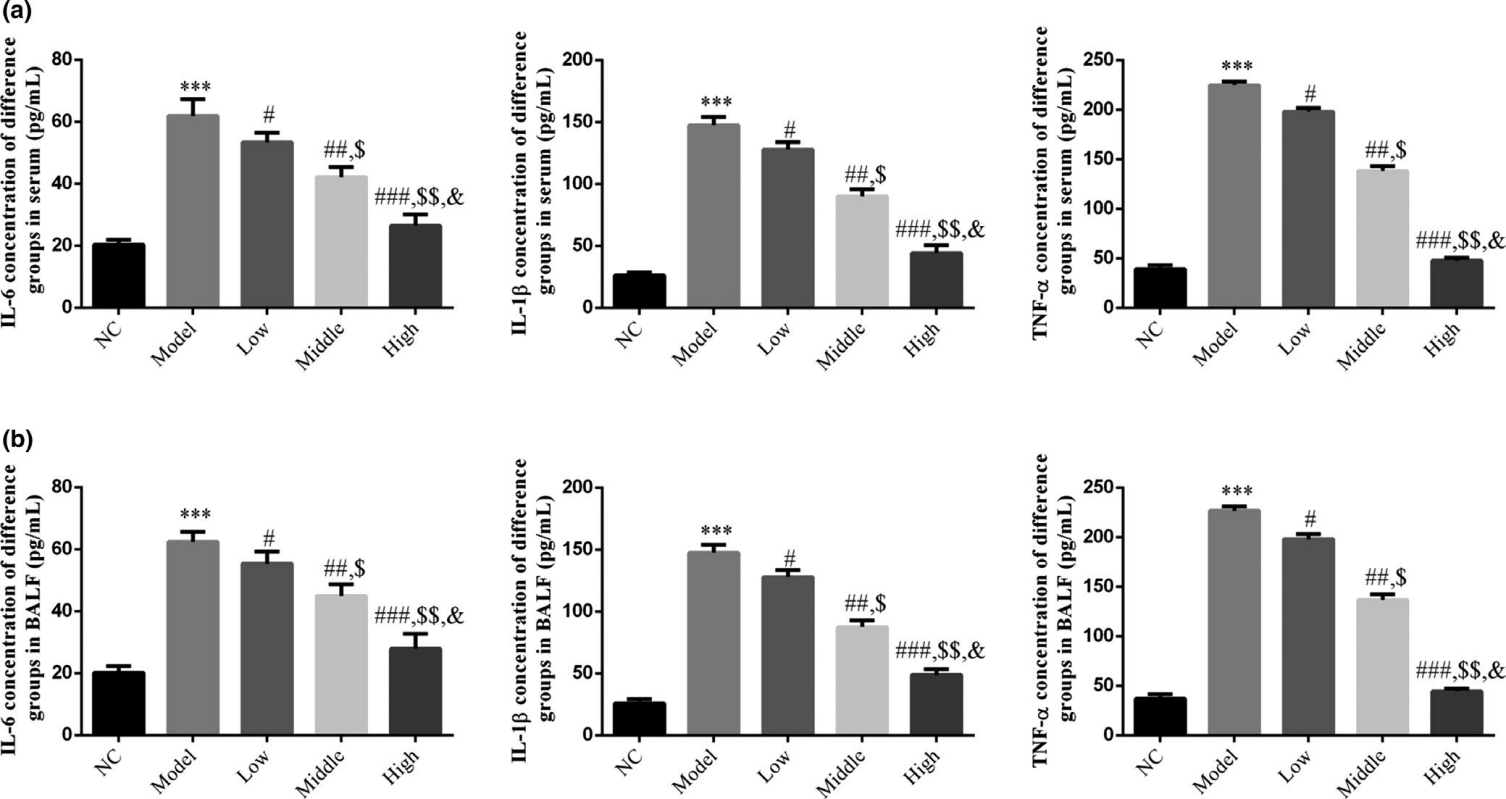
IL‐6, IL‐1β, and TNF‐α concentration of different groups by ELISA. NC: normal control rats; model: COPD rats; low: COPD rats treated with low‐dose houttuynia (5 mg/kg); middle: COPD rats treated with middle‐dose houttuynia (10 mg/kg); high: COPD rats treated with low‐dose houttuynia (5 mg/kg); middle: COPD rats treated with middle‐dose houttuynia (25 mg/kg). (a) IL‐6, IL‐1β, and TNF‐α concentration in serum. (b) IL‐6, IL‐1β, and TNF‐α concentration in BALF. ***: *p* < .001, compared with NC group; #: *p* < .05, ##: *p* < .01, ###: *p* < .001, compared with model group; $: *p* < .05, $$: *p* < .01, compared with low group; &: *p* < .05, compared with middle group

ELISA results showed that the contents of IL‐1β, IL‐6, and TNF‐α obviously increased in both the serum and BALF of the model group than in the NC group (*p* < .001; Figure 2a,b. After treatment with houttuynin, the contents of IL‐1β, IL‐6, and TNF‐α decreased in the serum and BALF of rats in the low, middle, and high groups (*p* < .001, Figure 2a,b and there was a significant dose–effect relationship among groups.

### 4.2 Effect of houttuynin on pathological morphology of lung tissue in COPD rats

**FIGURE 3:**
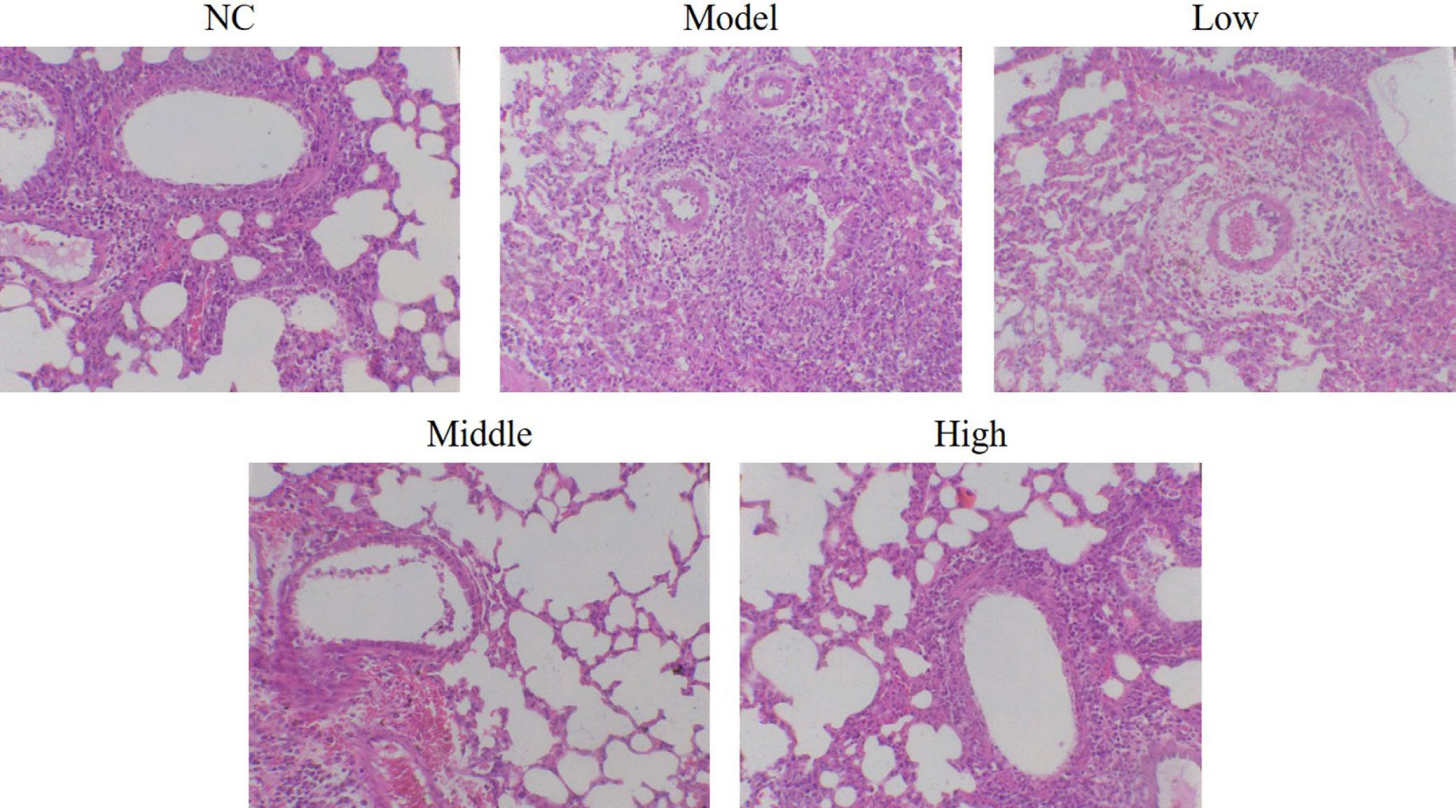
Effects of houttuynia on pathological morphology by HE staining (200×). NC: normal control rats; model: COPD rats; low: COPD rats treated with low‐dose Houttuynia (5 mg/kg); middle: COPD rats treated with middle‐dose houttuynia (10 mg/kg); high: COPD rats treated with low‐dose houttuynia (5 mg/kg); middle: COPD rats treated with middle‐dose houttuynia (25 mg/kg)

The lung tissue structure of rats in the NC group was basically intact, with no inflammatory cell infiltration or interstitial thickening. The model group demonstrated alveolar collapse, increased alveolar wall thickness, interstitial edema and congestion, inflammatory cell infiltration, fibroblast interstitial infiltration, and other histopathological changes. Meanwhile, the lung histopathological changes were significantly improved in the low, middle, and high groups, as shown in Figure 3.

### 4.3 Effect of houttuynin on apoptosis of lung tissue in rats with COPD

**FIGURE 4:**
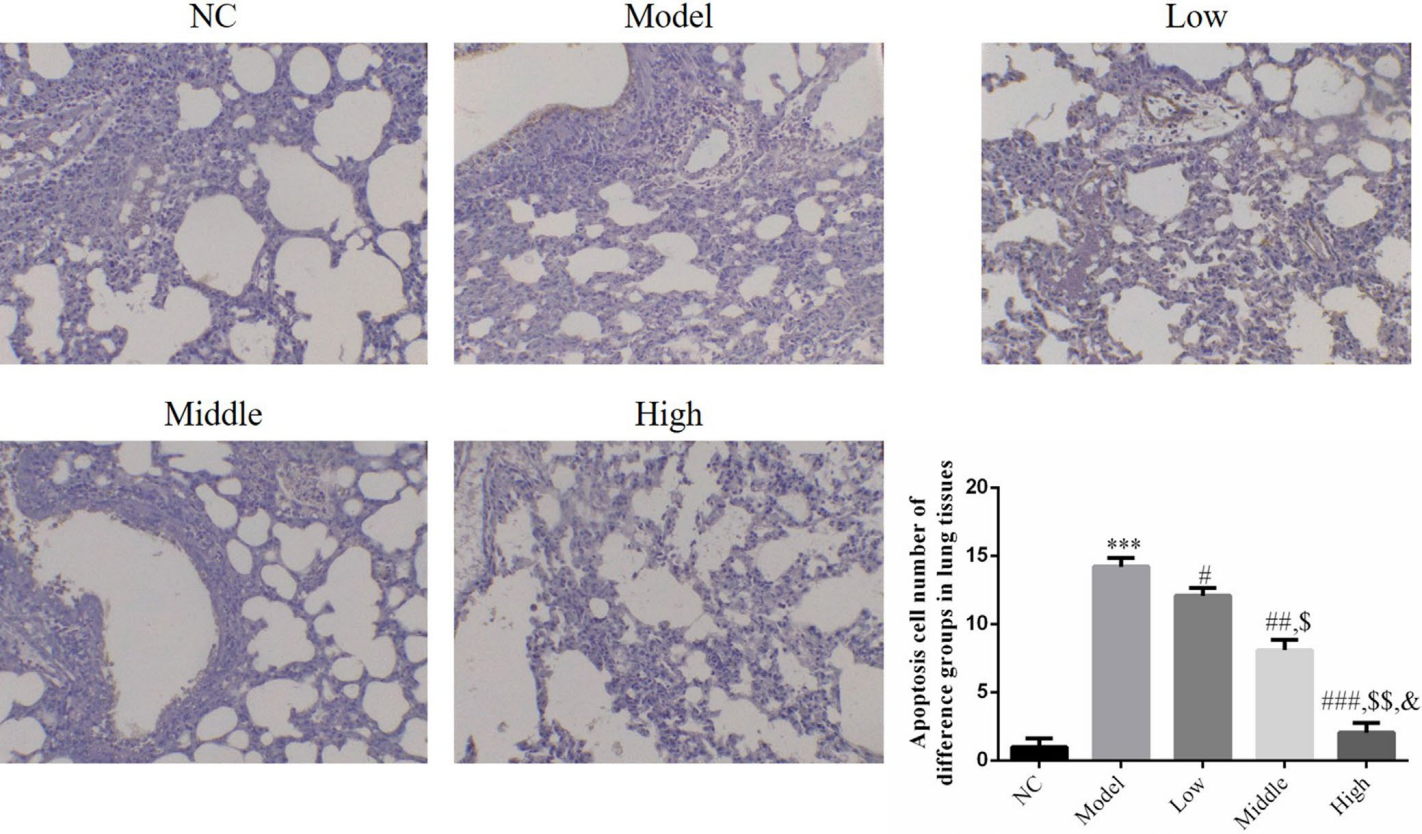
Apoptosis cell number of different groups by TUNEL assay (200×). NC: normal control rats; model: COPD rats; low: COPD rats treated with low‐dose houttuynia (5 mg/kg); middle: COPD rats treated with middle‐dose houttuynia (10 mg/kg); high: COPD rats treated with low‐dose houttuynia (5 mg/kg); middle: COPD rats treated with middle‐dose houttuynia (25 mg/kg). ***: *p* < .001, compared with NC group; #: *p* < .05, ##: *p* < .01, ###: *p* < .001, compared with model group; $: *p* < .05, $$: *p* < .01, compared with low group; &: *p* < .05, compared with middle group

There were a few apoptotic cells in lung tissues of rats in the NC group. Compared with the NC group, the number of apoptotic cells increased significantly in rat lung tissues of the model group (*p* < .001; Figure 3. Following intervention with houttuynin, the number of apoptotic cells in rat lung tissues of the low, middle, and high groups decreased significantly compared with that in the model group (*p* < .001, Figure 4, showing a dose–effect relationship.

### 4.4 Effect of houttuynin on the protein expression of caspase‐3 and caspase‐9 in lung tissue of rats with COPD

**FIGURE 5:**
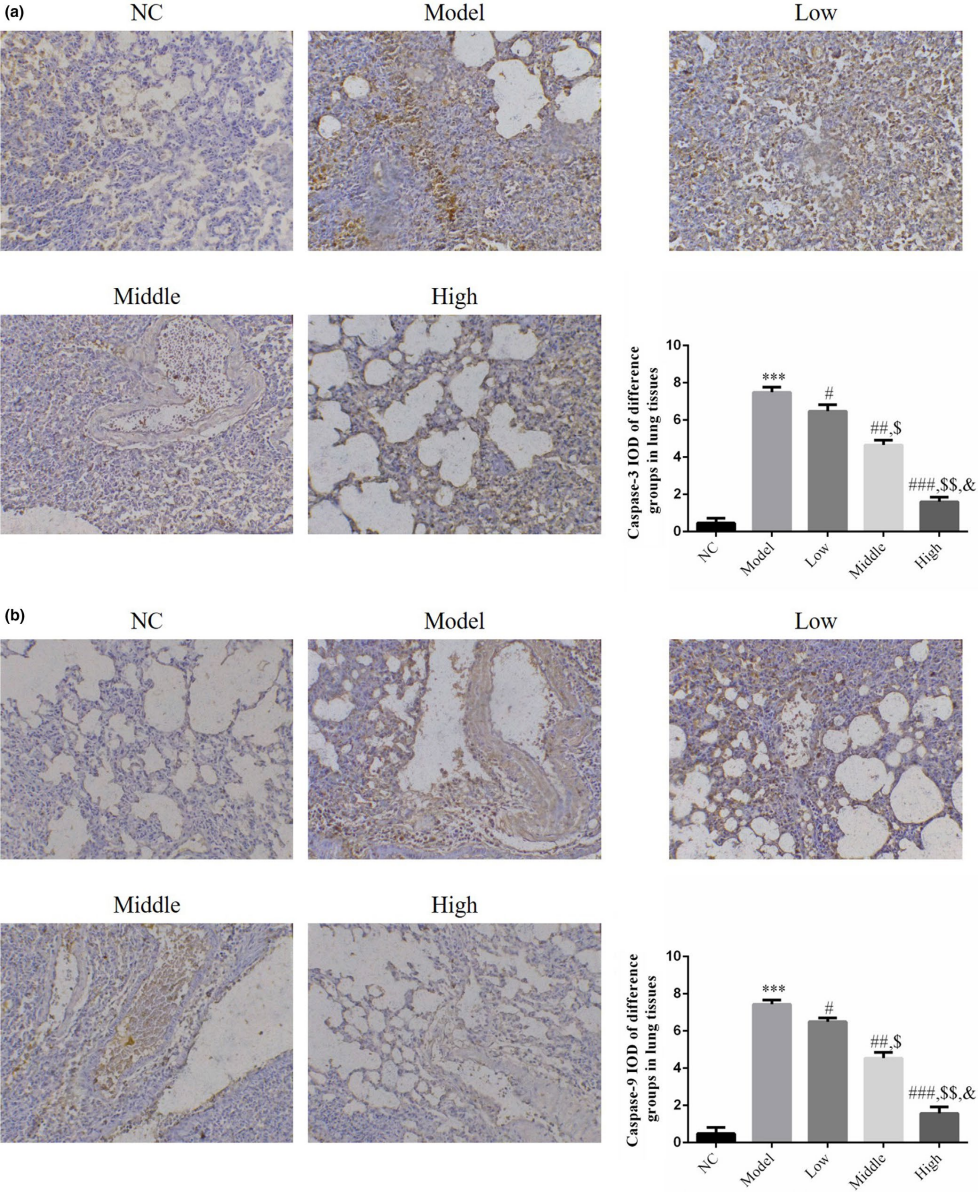
Apoptosis relative protein expression in different groups by IHC assay (200×). NC: normal control rats; model: COPD rats; low: COPD rats treated with low‐dose houttuynia (5 mg/kg); middle: COPD rats treated with middle‐dose houttuynia (10 mg/kg); high: COPD rats treated with low‐dose houttuynia (5 mg/kg); middle: COPD rats treated with middle‐dose houttuynia (25 mg/kg). (a) Caspase‐3 protein expression in different groups by IHC assay (200×). (b) Caspase‐9 protein expression in different groups by IHC assay (200×). ***: *p* < .001, compared with NC group; #: *p* < .05, ##: *p* < .01, ###: *p* < .001, compared with model group; $: *p* < .05, $$: *p* < .01, compared with low group; &: *p* < .05, compared with middle group

On the basis of the detection results of IHC, relatively low protein expression levels of caspase‐3 and caspase‐9 were observed in rat lung tissues of the NC group. Compared with the NC group, the protein expression levels of caspase‐3 and caspase‐9 significantly increased in rat lung tissues of the model group (*p* < .001; Figure 5a,b. Furthermore, compared with the model group, the protein expression levels of caspase‐3 and caspase‐9 in rat lung tissues of the low, middle, and high groups decreased significantly after houttuynin administration (*p* < .001,Figure 5a,b, exhibiting a dose–effect relationship.

### 4.5 Effect of houttuynin on the protein expression of TLR4, MyD88, and p‐NF‐κB(p65) in lung tissue of rats with COPD

**FIGURE 6:**
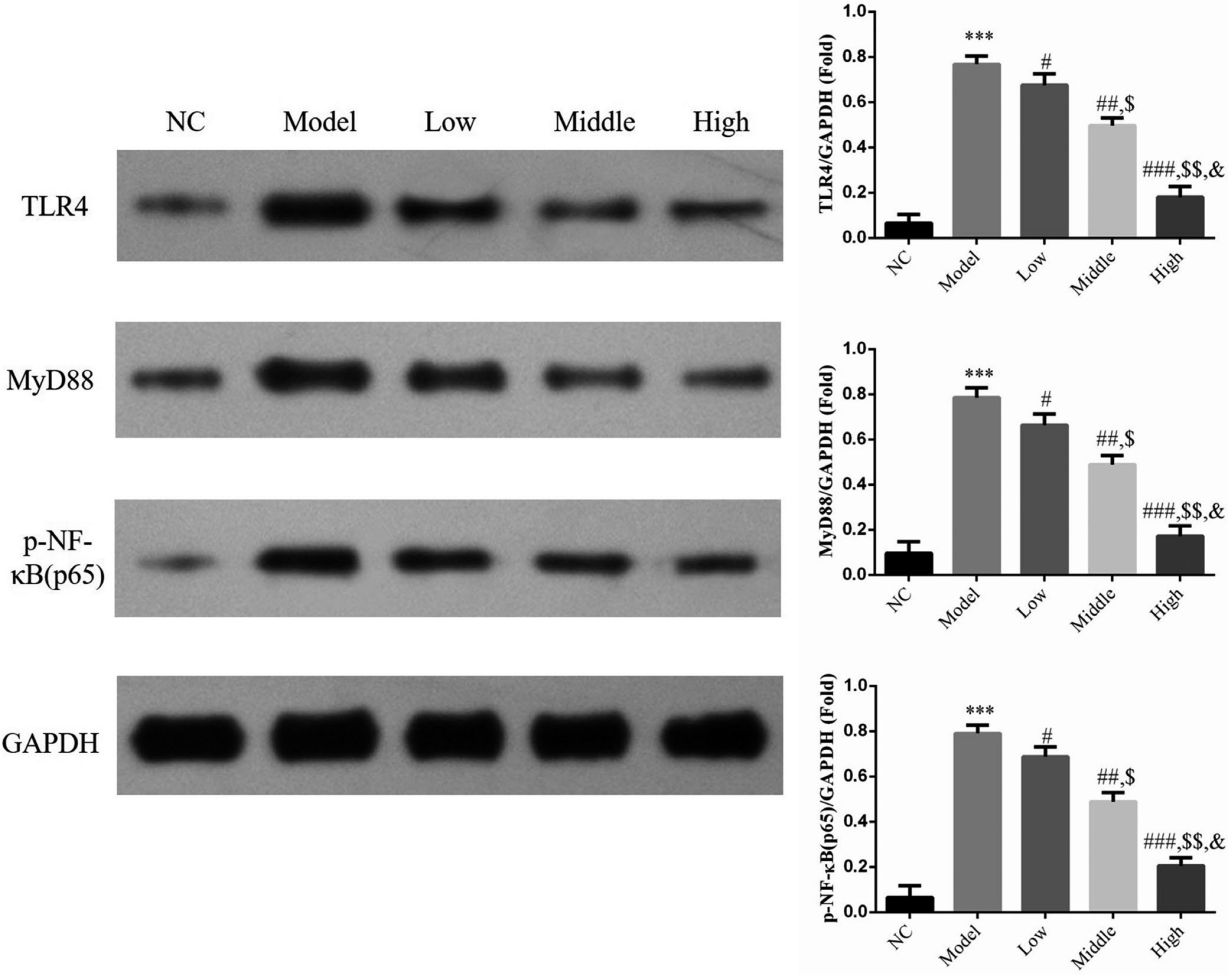
TLR4, MyD88, and p‐NF‐κB(p65) proteins expressions by WB assay. NC: normal control rats; model: COPD rats; low: COPD rats treated with low‐dose houttuynia (5 mg/kg); middle: COPD rats treated with middle‐dose houttuynia (10 mg/kg); high: COPD rats treated with low‐dose houttuynia (5 mg/kg); middle: COPD rats treated with middle‐dose houttuynia (25 mg/kg). ***: *p* < .001, compared with NC group; #: *p* < .05, ##: *p* < .01, ###: *p* < .001, compared with model group; $: *p* < .05, $$: *p* < .01, compared with low group; &: *p* < .05, compared with middle group

WB results revealed that the protein expression levels of TLR4, MyD88, and p‐NF‐κB(p65) were relatively low in rat lung tissues of the NC group. Compared with the NC group, the protein expression of TLR4, MyD88, and p‐NF‐κB(p65) increased in rat lung tissues of the model group (*p* < .001; Figure 5. With the administration of houttuynin, the protein expression of TLR4, MyD88, and p‐NF‐κB(p65) decreased in rat lung tissues of the low, middle, and high groups compared with those in the model group (*p* < .001, Figure 6, exhibiting a dose–effect relationship.

## 5 DISCUSSION

COPD is a disease with both high incidence and high mortality worldwide, with no definite mechanism elaborated thus far. At present, smoking and other behaviors are generally believed to induce abnormal inflammatory response of the lungs caused by harmful gases or particles, which is one of the pathogenesis of COPD. Therefore, in this study, the recognized method of cigarette smoking and LPS was adopted to induce the COPD animal model. The W/D ratio of rat lungs increased, accompanied by pathological damages (such as inflammatory infiltration and the development of fibrosis) and significantly increased secretion of inflammatory cytokines of TNF‐α, IL‐1β, and IL‐6 in lung tissues of rats with COPD. These findings were consistent with the results reported in the literature, suggesting the successful modeling of COPD in rats (Li et al., 2019, 2020). Similar to other inflammatory diseases, COPD is characterized by the accumulation and activation of inflammatory cytokines in the airway, which is essential to induce inflammation, including TNF‐α, IL‐1β, and IL‐6. Numerous studies have found that the expression of TNF‐α, IL‐1β, IL‐6, and other inflammatory mediators significantly increased, with enhanced activities of caspase‐3 and caspase‐9, which might jointly result in damaged lung function and elevated number of apoptotic cells in lung tissue (Yamada et al., 2016; Zhen et al., 2018). In accordance with the results of our study, houttuynin treatment reduced the protein expression of caspase‐3 and caspase‐9 and downregulated the expression of inflammatory cytokines of TNF‐α, IL‐1β, and IL‐6. Hence, houttuynin could effectively inhibit the release of inflammatory mediators caused by COPD, which explained the mechanism of lung injury at the molecular level.

NF‐κB(p65) is the most important transcription factor in regulating the inflammatory pathway. After phosphorylation and activation, NF‐κB(p65) can cause excessive secretion of inflammatory factors (Ma et al., 2020; Meng et al., 2020). Furthermore, the activation of TLR4 may further initiate MyD88 through a series of cascade reactions and then induce the activation of NF‐κB(p65) and transformation into the nucleus to bind with pro‐inflammatory gene promoters. These promoters may increase gene expression and expand inflammatory response, leading to inflammatory damage. Thus, the blockage of NF‐κB transcription activity may be a key target in the treatment of inflammatory diseases (Liu et al., 2014; Sun et al., 2014; Xu et al., 2016). The expression of TLR4 in peripheral blood of patients with COPD was obviously higher than that of normal subjects, and the NF‐κB signaling pathway exhibited an intimate association with smoking‐induced airway inflammation and COPD (Rom et al., 2013; Zhuan et al., 2017). In this study, the protein expression levels of TLR4, MyD88, and p‐NF‐κB(p65) increased in lung tissues of rats with COPD. Notably, houttuynin could inhibit the expression of the three indicators. Therefore, the response of COPD could be intervened by inhibiting the TLR4/MyD88 signaling pathway to improve pathological damage of the lungs.

In summary, houttuynin can reduce lung tissue congestion, edema, inflammatory cell infiltration, and collagen deposition and improve lung pathological damage. It can inhibit the expression of TNF‐α, IL‐1β, and IL‐6; downregulate the protein expression of caspase‐3 and caspase‐9; and inhibit the activation of the TLR4/MyD88/NF‐κB(p65) signaling pathway. Findings in this study have provided experimental data for houttuynin in the treatment of COPD.

## CONFLICT OF INTEREST

None.

## ETHICAL APPROVAL

This study was supported by Ethics committee of Wangjing Hospital.
